# Evaluation of the synapse adhesion molecule Kirrel3 in neurological disease

**DOI:** 10.3389/fneur.2025.1662931

**Published:** 2025-09-22

**Authors:** Omar Shennib, Olivia Raines, Amanda Sandoval Karamian, Megan E. Williams

**Affiliations:** ^1^Department of Neurobiology, School of Medicine, University of Utah, Salt Lake City, UT, United States; ^2^Division of Pediatric Neurology, Department of Pediatrics, School of Medicine, University of Utah, Salt Lake City, UT, United States

**Keywords:** gene disease association, genotype–phenotype correlation, clinical variant, neurodevelopmental disorders, autism, intellectual disability, cell adhesion molecule, Kirrel3

## Abstract

The synaptic adhesion molecule KIRREL3 regulates synapse development in mice and is implicated in human neurological disorders, including autism spectrum disorder, intellectual disability, and Jacobsen syndrome (chromosome 11q deletion syndrome). However, its status as a definitive human disease gene remains unresolved, likely due to the rarity of *KIRREL3*-related disorders and significant gaps in understanding its molecular mechanisms. Current knowledge is further fragmented across disparate clinical and basic research reports, often buried in supplemental data. This review synthesizes existing evidence to enable clinicians and scientists to better evaluate *KIRREL3* variants as potentially disease causing. We review its conserved role in mediating neuron-to-neuron interactions during axon targeting and synapse formation in mice and how disruptions to these interactions could contribute to neurological pathology in humans. We also discuss how disease-associated variants alter *KIRREL3* function. Our analysis underscores the need for integrated studies spanning basic and clinical investigation to validate *KIRREL3*’s disease association and advance future interventions for *KIRREL3*-related disorders.

## Introduction

*KIRREL3* is a member of the small IRM (Irregular cell recognition module) gene family and the only one repeatedly linked to neurological disorders (1–5). Like all IRM proteins, KIRREL3 is a single-pass transmembrane protein (1, 6). IRM proteins are best characterized in non-neural tissues, where they play critical roles in kidney slit diaphragm formation and muscle cell fusion (6–9). These proteins also contribute to neurodevelopmental processes in model organisms, including Drosophila eye formation and *C. elegans* synaptic connectivity (6, 7, 10–29). Because this important foundational work on invertebrate IRM proteins was reviewed elsewhere (30) and our goal is to translate *Kirrel3* knowledge to human neurological diseases, we focus this review on *Kirrel3* in brain function and dysfunction of mammals from mice to humans.

Over the past two decades, human genetic studies suggested associations between *KIRREL3* (OMIM: 607761) and brain conditions such as autism spectrum disorder, intellectual disability, Jacobsen syndrome, and developmental delay (4, 31–37). However, the role of *KIRREL3* as a disease-associated gene remains unresolved in the clinic even though there is supportive evidence of its variant pathogenicity in the scientific literature. Many different types of genetic variants in the *KIRREL3* gene, including missense mutations, truncating variants, splice-site alterations, and copy number variants, are reported in the public databases gnomAD, UCSC, VariCarta, and ClinVar (38–41). While some of these variants are classified as pathogenic or likely pathogenic, many others remain classified as variants of uncertain significance (VUS) due to insufficient evidence (41). This uncertainty stems from several factors, including small sample sizes in initial studies, limited functional validation, lack of inheritance information, and inconsistent phenotypic presentations among individuals with *KIRREL3* variants. Furthermore, the lack of large-scale, systematic analyses on *KIRREL3*-related disorders contributes to the unresolved role of *KIRREL3* in disease. This leaves clinicians and researchers without clear guidelines for interpretation.

The goal of this work is to provide a side-by-side analysis of what is currently known about KIRREL3 protein function in the mammalian brain, as well as what is known about Kirrel3 gene variants and their associations with neurological diseases. Furthermore, recent analyses also found increases *KIRREL3* gene expression to glioma cancer (32, 33). Misexpression of cell adhesion molecules is common in metastatic cancers, however, we will focus on neurological disorders in this review (42). This review will contribute to the understanding of *KIRREL3* and serve as a model for resolving gene-disease associations surrounding other genes with similarly unclear disease associations.

## KIRREL3 gene and isoforms expression

**Figure 1:**
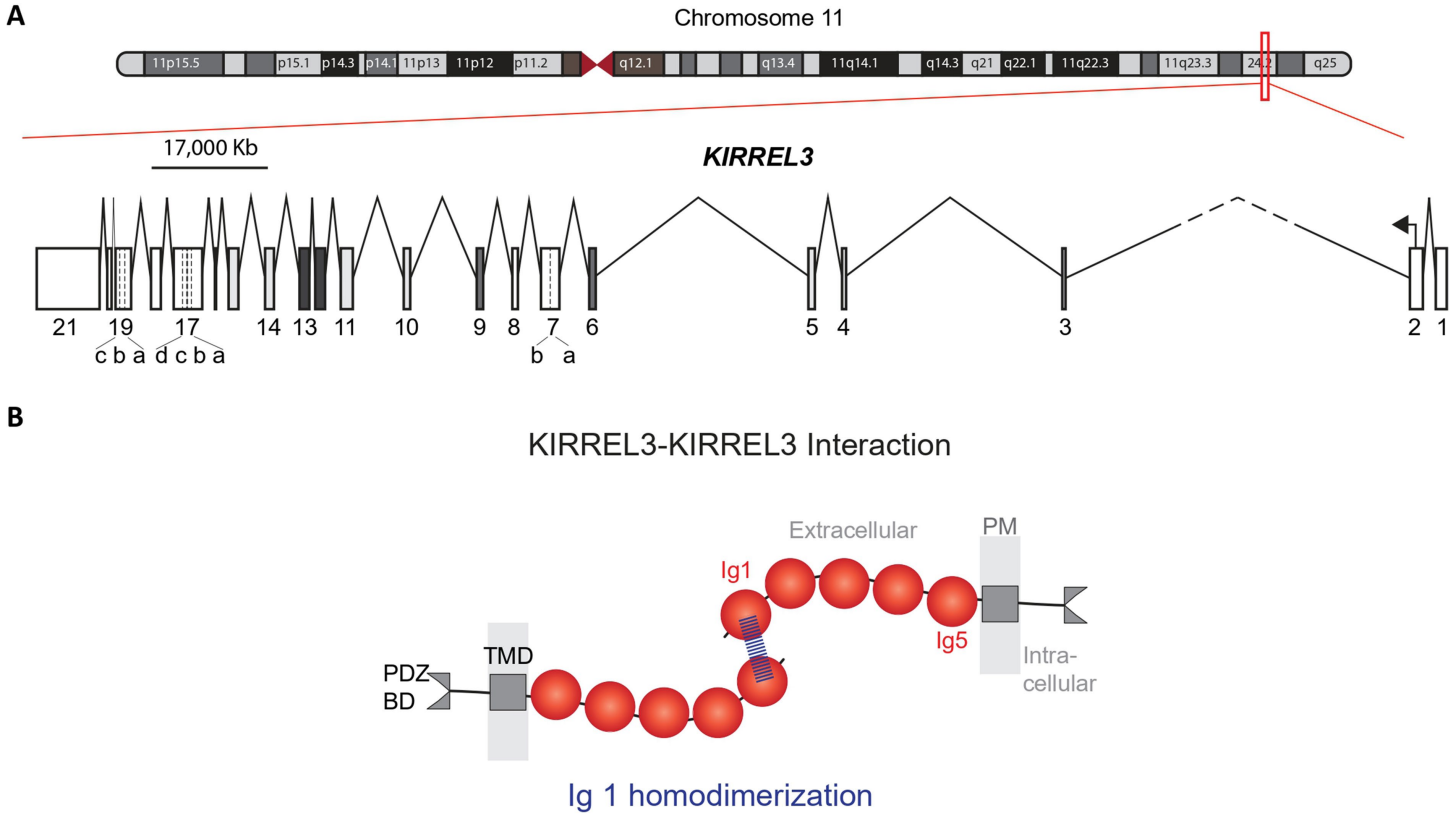
Human *KIRREL3* gene and protein structures. **(A)** Genetic map of chromosome 11. Red box covers the *KIRREL3* gene (577.413 Kb) on the q arm of chromosome 11. *KIRREL3* schematic gene structure shows 21 exons (boxes) and introns (lines). Exon 7, 17, and 19 are reported in unique isoforms and have 5′ extensions and 3′ extensions, indicated by dotted lines. Intron 2–3 is cut short (dashes) to fit in the scaled illustration. **(B)** KIRREL3 protein structure and protein transhomophilic interaction. Binding structural assay shows the protein binding structure is through the IgD1 via hydrogen bonding at Q128 amino acid position. IgD, immunoglobulin domain; TMD, transmembrane domain; BD, binding domain; PM, plasma membrane.

The human *KIRREL3* gene (Kin of IRRE-like 3), also previously known as *NEPH2* (43), is located on chromosome 11q24.2 (hg38 chr11:126,423,358-127,000,770), covering 577.413 Kb, with 21 exons (Figure 1A) (39, 44, 45). Its longest and most abundant transcript encodes a single-pass transmembrane protein that is 778 amino acids long with five extracellular immunoglobulin domains and an intracellular region ending in a PDZ binding domain (Figure 1B) (44, 46, 47). Targeted long-read mRNA sequencing revealed the full exon structure and alternative splicing pattern of *Kirrel3* in the mouse brain (45). In mice, 19 alternatively spliced isoforms were identified including those with different intracellular domains and secreted forms. The study also analyzed publicly available long-read sequencing data from human brain tissue and identified 11 isoforms in the human brain (45). Although, this number is likely an underestimate because *Kirrel3* transcripts are rare and a targeted approach was not used, the patterns were similar to mice in that human *KIRREL3* isoforms also had different intracellular domains and secreted forms (45). Interestingly, cross-species comparisons of *Kirrel3* transcripts revealed that humans and great apes uniquely possess an additional exon encoding a distinct 30 amino acid insertion in the intracellular domain, suggesting evolutionary specialization (45). However, future work is needed to directly test if different *KIRREL3* isoforms, including the hominid-specific exon, have distinct functions in the brain.

**Figure 2:**
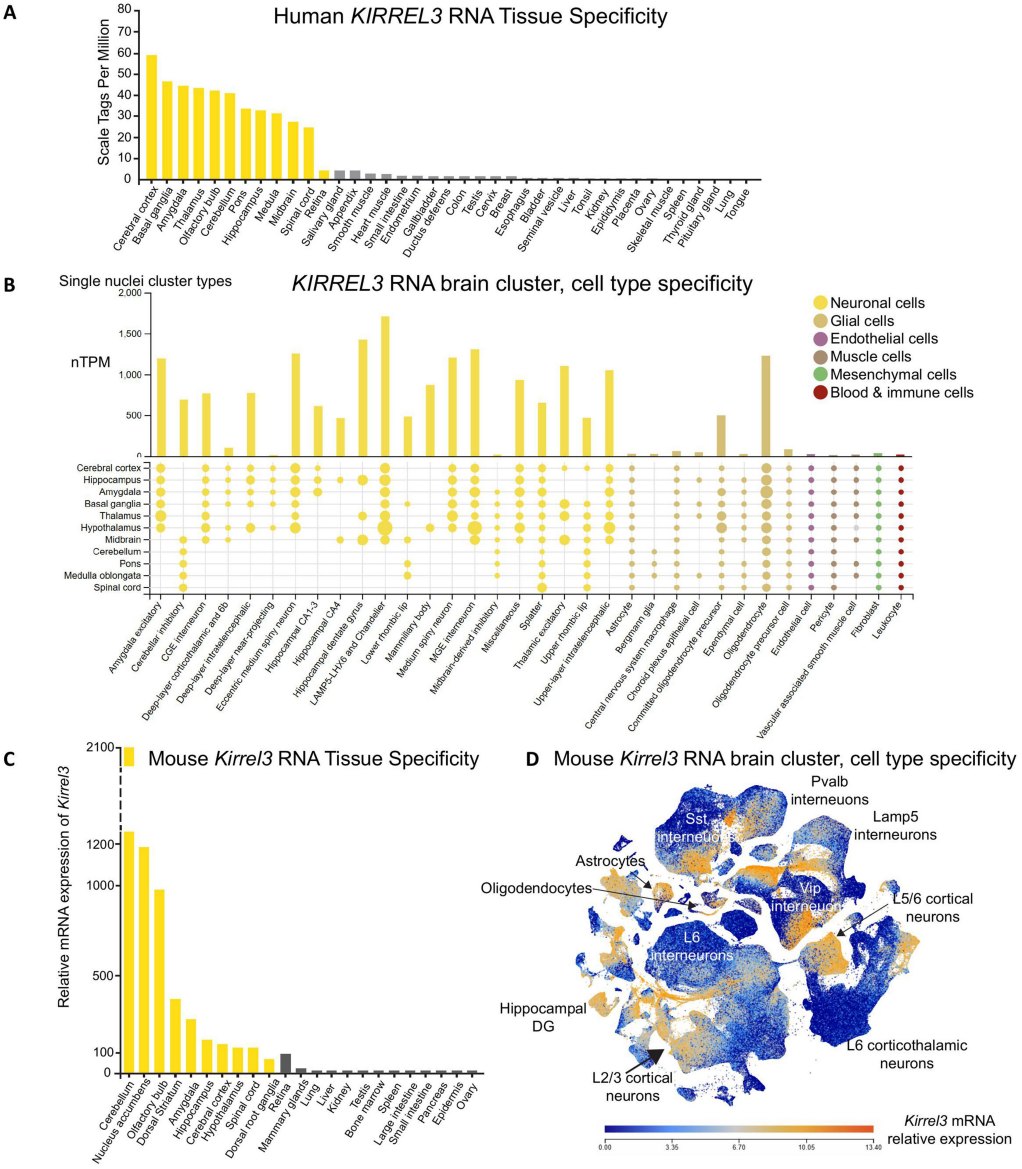
*KIRREL3* expression in human and mouse. **(A)** RNA sequencing from adult human tissues show *KIRREL3* mRNA expression levels higher in brain regions (yellow) than anywhere else. **(B)** Single nuclei sequencing from human brain shows that *KIRREL3* copies are enriched in excitatory and inhibitory neurons, as well as gliodendrocytes. Y-axis shows transcript expression in normalized transcript per million (nTPM). Human transcript expression levels are based on transcriptomics data from two sources: HPA and GTEX. **(C)** Mouse *Kirrel3* mRNA expression is similar to human with high expression in brain regions (yellow). **(D)** Scatter blot of mouse *Kirrel3* transcript expression shows expression in specific cell types throughout the brain. Mouse transcriptomics data are from two sources: BioGFP and Allen Brain Atlas.

The *KIRREL3* mRNA is expressed in multiple tissues, including the kidney, skeletal muscle, and brain (16, 47, 48). To refine its expression profile using recent datasets, we analyzed RNA-seq and single-nucleus RNA sequencing available on GTEx, Human Protein Atlas, BioGPS, and Allen Brain Atlas (46, 49–51). We found that both human and mouse *Kirrel3* mRNA have the highest expression in the brain with enrichment in the cerebral cortex, thalamus, cerebellum, amygdala, hippocampus, and olfactory bulb (Figures 2A,C). Within the brain, *Kirrel3* transcription is enriched in three major cell populations: excitatory neurons (amygdala and hippocampal dentate gyrus (DG)), inhibitory neurons (LAMP5-LHX6, Chandelier, and MGE-derived interneurons), and oligodendrocytes (Figures 2B,D). The conserved expression pattern of *Kirrel3* in both human and mouse brain cell types, and the high conservation in the gene (94.7%) and protein (98%) sequences, supports the use of mouse models to elucidate its cellular function (52–55).

## KIRREL3 behavioral studies in mouse

Several mouse models were developed to investigate *Kirrel3*’s role in neurological disorders, including a global knockout, a conditional knockout, and a point mutant knock-in mouse. *Kirrel3* knockout mice were used to investigate behavioral phenotypes relevant to neurological disorders and compared to wildtype controls (56–59). *Kirrel3* knockout mice exhibit subtle deficits in social recognition and novel object memory, which may be consistent with core features of autism spectrum disorder and intellectual disability in humans (56, 58). Moreover, a recent study revealed that selective activation of *Kirrel3*-expressing GABAergic neurons in the hippocampus impairs contextual memory discrimination, directly linking *Kirrel3*-expressing GABA neurons to cognitive processes (59).

Beyond memory functions, *Kirrel3* knockout mice show distinct behavioral alterations across multiple neural systems. While basic olfactory function remains intact, knockout mice show reduced male–male aggression (56, 57, 60). Motor assessments yield complex findings, with knockout mice displaying both hyperactivity in open field tests and enhanced coordination on rotarod tasks (56–58, 61). Auditory system evaluation reveals a selective deficit in processing high-intensity sounds (110 dB), while baseline hearing remains unaffected (57). Though molecular mechanisms were investigated in hippocampal and olfactory circuits (See Molecular Mechanisms Section), significant gaps remain in understanding *Kirrel3*’s precise function in other brain regions. The lack of mechanistic studies in cerebellar and auditory circuits limits our interpretation of the observed behavioral phenotypes. Collectively, *Kirrel3* knockout behavioral studies indicate that *Kirrel3* subtly influences distinct brain functions in mice, including cognition, olfaction, motor control, and auditory processing. These functions are clinically relevant, as they are commonly disrupted in autism and intellectual disability (62–64). However, a caveat of mouse behavioral studies is that they were done in adult mice and genetic compensation can occur over time. More behavioral assays in juvenile mice are needed to directly assess the developing brain and ameliorate this caveat. Future research using conditional knockout models would clarify *Kirrel3*’s region-specific effects on mouse brain function. Additionally, although automated tracking methods are propelling the complexity of mouse behavioral research, mice inherently lack the subtle and complex behaviors of humans and direct translation from mouse to human should be interpreted with caution.

## Molecular mechanisms of KIRREL3 in mouse

In general, *Kirrel3* knockout mice exhibit normal brain and body size throughout development compared to wildtype controls (56–58). Macroscopic-level findings show that *Kirrel3* manipulation does not alter gross neuroanatomy but instead affects ultrastructures (52, 56–58, 60, 65, 66). This is similar to autism where there is no clear or consistent pathology at a macroscopic structural level, but it is largely theorized that deficits are at a synaptic and circuit level (63, 67). Thus, we next review mechanistic studies of *Kirrel3* in the mammalian brain.

### Kirrel3 promotes hippocampal synapse formation

**Figure 3:**
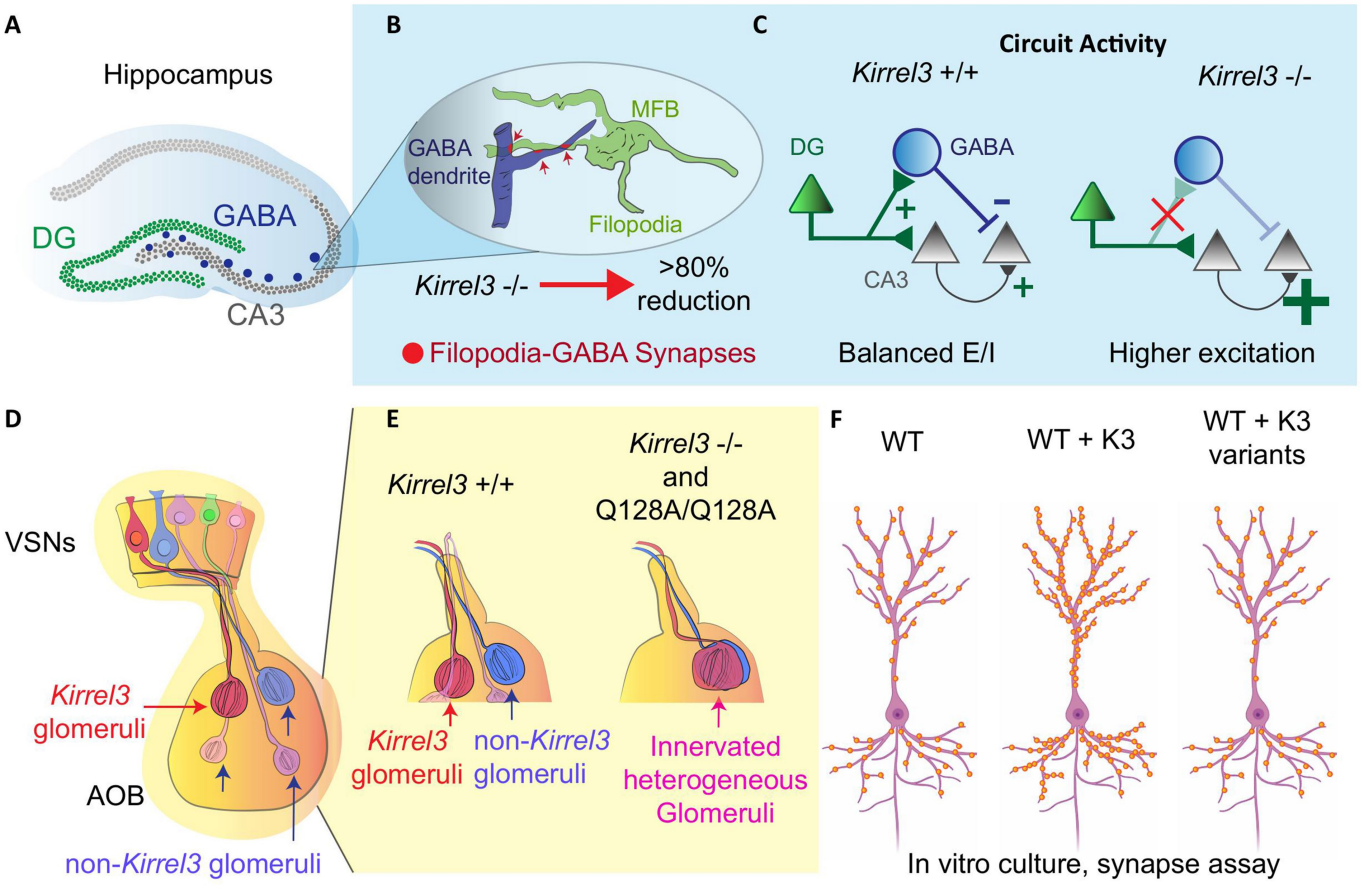
*Kirrel3* functional studies in rodents. **(A)** A Hippocampal diagram and its relevant regions (DG, CA3 and GABA neurons). **(B)** Structure of mossy fiber botouns (MFB) and filopodia-GABA synapses in the CA3 region. In the *Kirrel3* KO mouse, these synapses are reduced significantly. **(C)** CA3 neurons have higher excitation/inhibition ratio (E/I) in knockout mice. This is most likely due to the loss of feed-forward inhibition from the DG filopodia-GABA synapse reduction. **(D)** An olfactory system diagram shows vomeronasal sensory neurons (VSN) projecting axons to form synaptic *Kirrel3* homogeneous glomeruli in the accessory olfactory bulb (AOB). **(E)** *Kirrel3* KO and point mutant (Q128A) mice show overall a reduction in glomeruli number compared to wild-type (WT) mouse. **(F)** *In vitro* hippocampal culture, over expressing *Kirrel3* (K3) patient variants shows similar synapse number to the no overexpression condition in the WT. Only over-expression of *Kirrel3* wild-type copy increases the synapse number. This indicates that the *Kirrel3* patient variants alter *Kirrel3’s* synaptic function.

*Kirrel3* is expressed selectively in two hippocampal cell populations: excitatory dentate gyrus (DG) neurons and a subset of inhibitory GABAergic neurons (47, 52, 58). *Kirrel3* is important for the formation of specialized excitatory synapses onto GABAergic neurons in mice called mossy fiber filopodia synapses (Figures 3A,B) (52, 66). *Kirrel3* knockout mice have a significant reduction in the formation of mossy fiber filopodia synapses (66). Because these synapses normally excite GABAergic neurons that, in turn, inhibit CA3 neurons, *Kirrel3* knockout mice lack this inhibition and have a corresponding increase in CA3 neuron activity (Figure 3C) (52). Thus, a reduction in excitatory synapses actually increases net brain activity (because they specifically form on inhibitory neurons) and highlights why understanding the precise synapses regulated by neurodevelopmental risk genes is important for developing effective treatment strategies. Unlike disorders involving general excitatory synapse loss, where therapies might aim to boost neuronal activity, *KIRREL3*-related dysfunction may instead require interventions that decrease neuronal activity.

A subsequent study did not directly study KIRREL3 protein function but instead studied the role of the GABA neurons that express *Kirrel3* in learning, memory, and circuit activity (59). When these *Kirrel3*-expressing GABA neurons were activated with chemogenetics, they impaired memory and powerfully suppressed CA3 activity (59), confirming their capacity for circuit inhibition. However, it remains untested whether such activation could rescue the hyperactivity phenotype in knockout mice, a crucial next step for therapeutic development. These results are particularly significant given the established role of GABAergic dysfunction in autism spectrum disorders (68). Importantly, *Kirrel3*’s expression pattern extends far beyond the hippocampus, and is expressed by specific cell types in many brain regions (Figure 2) (46, 49, 51). Comparative studies across brain regions are needed to test whether *Kirrel3*’s hippocampal mechanisms generalize to other areas.

### Kirrel3 axon coalescence role in the olfactory system

Within the olfactory system, *Kirrel3* is expressed in discrete subsets of vomeronasal sensory neurons that converge to form homogeneous glomeruli in the accessory olfactory bulb (Figure 3D) (48, 60, 69, 70). This restricted expression implicates *Kirrel3* in helping to establish part of the glomerular map essential for odor and pheromonal processing. *Kirrel3* knockout mice display defects in posterior accessory olfactory glomeruli, which appear disorganized and significantly reduced in number compared to wildtype controls (Figure 3E) (48, 60). KIRREL3 is known to mediate synapse formation and cell adhesion through homophilic binding in trans where the KIRREL3 extracellular domain from one cell binds the KIRREL3 extracellular domain on another cell synapses (2, 6, 48, 52, 65, 71, 72). This was shown to occur *in vivo* through study of a p.Q128A point mutant that breaks the KIRREL3 homophilic trans-cellular binding (71). The mutant mouse phenocopied the glomeruli disruption in knockout mice and microscopic investigation showed that these glomeruli are innervated and heterogeneous (Figure 3E) (71). This indicates that KIRREL3-KIRREL3 trans-cellular binding is necessary for its function in the mouse brain. Olfactory deficits in *KIRREL3*-related disorders remain understudied, and this wiring mechanism might have broad relevance. The accessory olfactory bulb shares developmental pathways with social brain circuits, and disrupted pheromone processing could contribute to the social behavior impairments seen in patients.

## KIRREL3 variants in human neurological disorders

*KIRREL3*-related disorders have broad phenotypic spectrum and variable symptom severity. Affected individuals present with neurodevelopmental conditions, including autism, intellectual disability, developmental delay, ADHD, or bipolar disorder, often accompanied by dysmorphic facial features, e.g., coarse facies, large ears, and flat nasal bridge (Table 1). Moreover, symptom severity ranges from mild to profound. We explore specific variants and severity correlations in a later section (SNV in clinical cases and Table 2). The link between *KIRREL3* and neurological disorders was first discovered through two foundational studies. Grossfeld et al. (73) first identified deletions encompassing *KIRREL3* in patients with 11q terminal deletion syndrome. Subsequently, Bhalla et al. (31) made two key discoveries. First, they identified deletion variants in *KIRREL3*, including a balanced translocation. This chromosomal translocation disrupted both *KIRREL3* and *CDH15* in a severely affected female patient with an IQ of 16. Second, they reported the first pathogenic single nucleotide variants in *KIRREL3*. These included three missense mutations, p.R40W, p.R336Q, p.V731F, found in unrelated intellectual disability patients (31, 74). These three variants showed rare allele frequencies (0.0000016–0.000075) suggesting negative selection, and subsequent functional studies confirmed their impairment of synapse formation *in vitro* (2).

**Table 1:** Manual curation of well-reported exonic variants.

Publications	Transcript change	aa change	Variant type	Inheritance	Diagnosis	Individual ID	Sex	CADD score
Hu et al. (79)	C > T	Q426*	nonsense	P	ASD	14	M	49
De Rubeis et al. (78), on SFARI	C > T	Q259*	nonsense	Unk	ASD	Unknown	Unk	45
Stessman et al. (80)	C > T	Q259*	nonsense	M	ASD	TASC_217–14123-2170	Unk	45
Stessman et al. (80)	C > T	R756*	nonsense	M	ASD	Antwerp_82609	Unk	39
Guo et al. (81)	C > T	A393V	missense	M	ASD	M20306	M	36
Guo et al. (81)	C > T	P446L	missense	M	ASD	M16257	M	36
Leblond et al. (82)	C > A	E562L	missense	Both	mild ASD, normal IQ	PN400116	F	35
Bhalla et al. (31)	C > T	R40W	missense	De	Sever ID (IQ < 50)	CMS7706	Unk	35
Stessman et al. (80)	C > T	R40W	missense	De	ASD	Leiden_D1.06.06912	Unk	35
Stessman et al. (80)	C > T	R513W	missense	Unk	ASD	Gecz_17_2072	Unk	35
Leblond et al. (82)	C > A	R562L	missense	Both	mild ASD, normal IQ	PN400528	F	35
Zhou et al. (75)	G > A	R566Q	missense	De	ASD	SP0314188	F	35
Stessman et al. (80)	G > A	G360S	missense	De	ASD	Gecz2_34806	Unk	34
Stessman et al. (80)	G > A	R687C	missense	P	ASD	14071	Unk	34
Stessman et al. (80)	G > A	V331I	missense	Unk	ASD	Antwerp_103567	Unk	34
Stessman et al. (80)	G > A	C398F	missense	Unk	ASD	TASC_214–17025-1	Unk	33
Guo et al. (81)	C > A	Q428K	missense	P	ASD	M19544	M	33
Stessman et al. (80)	C > T	R490W	missense	Unk	ASD	TASC_217–14183-2990	Unk	33
Stessman et al. (80)	G > A	R650H	missense	Unk	ASD	SAGE_330.03	Unk	33
Guo et al. (91)	G > A	R662H	missense	De	ASD, Learning Disability	4170.03	M	33
Stessman et al. (80)	G > A	R682Q	missense	Unk	ASD	Gecz_18_2326	Unk	33
Stessman et al. (80)	C > T	R694Q	missense	P	ASD	11657	Unk	33
Stessman et al. (80)	G > A	V556M	missense	Unk	ASD	Antwerp_108448	Unk	32
Stessman et al. (80)	G > T	D678Y	missense	Unk	ASD	Troina2_04233–9,007	Unk	31
De Rubeis et al. (78)	G > A	R205Q	missense	De	ASD2	1424JS0003	M	31
Stessman et al. (80)	G > A	R336W	missense	M	ASD	13918	Unk	31
Stessman et al. (80)	C > T	R389H	missense	P	ASD	14551	Unk	31
Guo et al. (81)	G > A	A393T	missense	P	ASD	M26986	M	30
Kalsner et al. (5)	G > A	A393T	missense	M	ASD, DD, see Table 2 for more	Unknown	Unk	30
Kalsner et al. (5)	G > A	A393T	missense	M	ASD, DD, see Table 2 for more	Unknown	Unk	30
Ciaccio et al. (83)	C > T	S255L	missense	De	ASD, Severe ID (GQ 24), see Table 2 for more	NA	M	30
Zhou et al. (75)	G > A	R161H	missense	De	ASD	12644.p1	M	29.5
Zhou et al. (75)	G > A	R161H	missense	De	ASD, level 2	08C78417	M	29.5
Li et al. (77)	G > T	W358C	missense	Familial	ASD	27844	Unk	29.3
Kalsner et al. (5)	G > T	K522N	missense	P	ASD	Unknown	Unk	29.2
Trost et al. (76)	G > A	R161H	missense	De	ASD, level 2	SSC06043	Unk	29.1
Kalsner et al. (5)	G > C	E411Q	missense	M	ASD, DD, see Table 2 for more	Unknown	Unk	27.6
Bhalla et al. (31)	G > A	R336Q	missense	De	mild ID (IQ > 50),seizures	Unknown	Unk	27.6
Bhalla et al. (31)	G > A	R336Q	missense	De	Sever ID (IQ < 50)	CMS8027	Unk	27.6
Bhalla et al. (31)	G > A	R336Q	missense	De	Sever ID (IQ < 50)	Unknown	Unk	27.6
De Rubeis et al. (78)	A > G	E581G	missense	Pl	ASD	DEASD_0166_001	M	26.2
Querzani et al. (3)	G > C	R668P	missense	De	mild ID, see Table 2 for more	NA	M	26.1
Hildebrand et al. (84)	G > T	S729I	missense	Unk	ASD	Unknown	Unk	25.5
Li et al. (77)	C > T	S241L	missense	Familial	ASD	17599	Unk	25.2
De Rubeis et al. (78), on SFARI	G > A	V30M	missense	P	ASD	Unknown	Unk	25.2
Kalsner et al. (5)	C > T	T413I	missense	P	ASD, DD, See Table 2 for more	Unknown	Unk	23.8
Taylor et al. (2)	G > A	M673I	missense	De	Global DD, moderate ID, see Table 2 for more	NA	M	23.7
De Rubeis et al. (78), on SFARI	T > C	V303A	missense	Unk	ASD	Unknown	Unk	23.2
De Rubeis et al. (78), on SFARI	G > C	K745N	missense	Unk	ASD	Unknown	Unk	23
Bhalla et al. (31)	G > A	V731I	missense	De	mild ID (IQ > 50)	CMS7941	Unk	21.9
Xin et al. (92)	T > G	F213V	missense	M	ASD	7714	M	21
Zhou et al. (75)	T > C	I208V	missense	De	ASD	GDX_57040	M	18.5
Kalsner et al. (5)	G > T	V219L	missense	P	ASD	Unknown	Unk	14.7
Deciphering Developmental Disorders Study (86)	C > T	T567M	missense	De	DD	DDD4K.01690	F	13.1
De Rubeis et al. (78)	C > G	H171Q	missense	M	ASD	Unk	Unk	1.1

P, Parental; M, Maternal; De, De novo; Unk, Unknown; M, Male; F, Female; NA, Non-Applicable; aa, amino acid.

**Table 2:** *KIRREL3* variants reported with detailed clinical manifestation.

aa change	Clinical manifestation	Publications
A393T	ASD, DD, dysmorphisms (coarse face, flat nasal bridge), anemia, central apnea	Kalsner et al. (5)
A393T	ASD, DD, dysmorphisms (coarse face, large ears, prominent jaw), anemia. Brain MRI: Chiari I malformation	Kalsner et al. (5)
E411Q	ASD, DD, dysmorphisms (epicanthus, flat nasal bridge), allergies	Kalsner et al. (5)
F213V	ASD, Bipolar disorder, Insomnia, Scoliosis, Chronic pain, Recurrent fractures, Intussusception, Pyloric stenosis	Xin et al. (92)
K522N	ASD, DD	Kalsner et al. (5)
M673I	Moderate ID, Global DD, ADHD, and obesity. Neonatal hypotonia and fine motricity deficits, mild dysmorphic features (i.e., exophthalmos, bulbous nose with slightly anteverted nares, thin upper lip, and wide-spaced teeth with two missing superior canines). Mild temporal cortical atrophy and wide asymmetric aspect of posterior ventricular horns.	Taylor et al. (2)
R161H	ASD 2	Trost et al. (76)
R205Q	ASD 2	De Rubeis et al. (78)
R336Q	mild ID (IQ > 50), Seizures	Bhalla et al. (31)
R336Q	Severe ID (IQ < 50), <25% head circumference, dysmorphic face	Bhalla et al. (31)
R336Q	Severe ID (IQ < 50), <25% head circumference, dysmorphic face	Bhalla et al. (31)
R40W	Severe ID (IQ < 50), <25% head circumference	Bhalla et al. (31)
R562L	mild ASD, normal IQ	Leblond et al. (82)
R662H	ASD, Learning Disability	Guo et al. (91)
R668P	Mild ID, cerebellar anomalies (cerebellar hypoplasia and mega cisterna magna) and minor dysmorphic features. (IQ: 75–77). Asymptomatic elevation of serum creatine phosphokinase (CPK) levels up to twice the normal value, range 219–315 mg/dL. mild dysmorphic features: long eyelashes, slightly anteverted nostrils, long nasal philtrum, mild asymmetric pectus excavatum	Querzani et al. (3)
S255L	ASD, Severe ID (GQ 24), muscular hypotonia, mildly ataxic gate, dysmorphisms (high-nasal bridge, long philtrum, and mild lower lip eversion), long fingers EEG: frontal epileptic anomalies. Brain MRI: mild cerebellar hypoplasia, mega cisterna magna	Ciaccio et al. (83)
T413I	ASD, DD, dysmorphisms (epicanthus, flat nasal bridge). Brain MRI: Chiari I malformation	Kalsner et al. (5)
V219L	ASD, DD	Kalsner et al. (5)
V731I	Mild ID (IQ > 50)	Bhalla et al. (31)

ASD2, Autism spectrum disorder level 2; patients require substantial support unlike ASD1 patients; ID, Intellectual disability; DD, Developmental delay; IQ, Intelligence quotient; GQ, Growth quotient; ADHD, Attention-Deficit/Hyperactivity Disorder; aa, amino acid.

### Testing the function of human variants in rodent neurons

*KIRREL3* missense variants identified in patients with autism and intellectual disability were functionally tested in a series of experiments using cultured cells and mouse neurons (2). Exogenous expression of wildtype *Kirrel3* traffics to the cell membrane, accumulates at synapses, causes cells to adhere to one another, and induces extra synapses in cultured mouse hippocampal excitatory neurons (2). Six variants were tested and trafficked correctly (R40W, R161H, R205Q, R336Q, M673I, and V731F); however, five out of the six variants, excluding M673I, had a reduced ability to induce synapses in cultured hippocampal neurons (Figure 3F) (2). Two variants that were not synaptogenic, p.R205Q and p.V731F, were unable to mediate trans-cellular adhesion, explaining their inability to mediate synapse formation (2). However, p.R40W, p.R161H and p.R336Q variants were not synaptogenic but could mediate cell adhesion normally (2). Together, this indicates that KIRREL3 trans-cellular binding is necessary but not sufficient for its synaptic function. This provides strong support that KIRREL3 is more than just synaptic glue and is also an active molecule in synapse formation. To our knowledge, this is the only study to date to investigate the function of *KIRREL3* missense variants found in patients with neurodevelopmental disorders. The fact that most disease-associated variants (5/6) have a clear functional deficit in cell and neuron culture assays strongly suggests that they may contribute to pathogenicity in humans.

### SNVs in clinical cases

To gain a better picture of *KIRREL3* SNV types from a clinical database, we analyzed 116 clinically validated *KIRREL3* variants from ClinVar (41). Human *KIRREL3* gene and transcript (NM_032531.4) references are annotated by the NCBI *Homo sapiens* Annotation Release 109.20200815; however, for the newly discovered exons, we used the most recent published data (45). For allele and variant frequencies, we used gnomAD Joint Variant Frequencies 4.0 v2 (74). We calculated CADD score, if not reported already, for variants in Table 1 using https://cadd.gs.washington.edu/snv.

**Figure 4:**
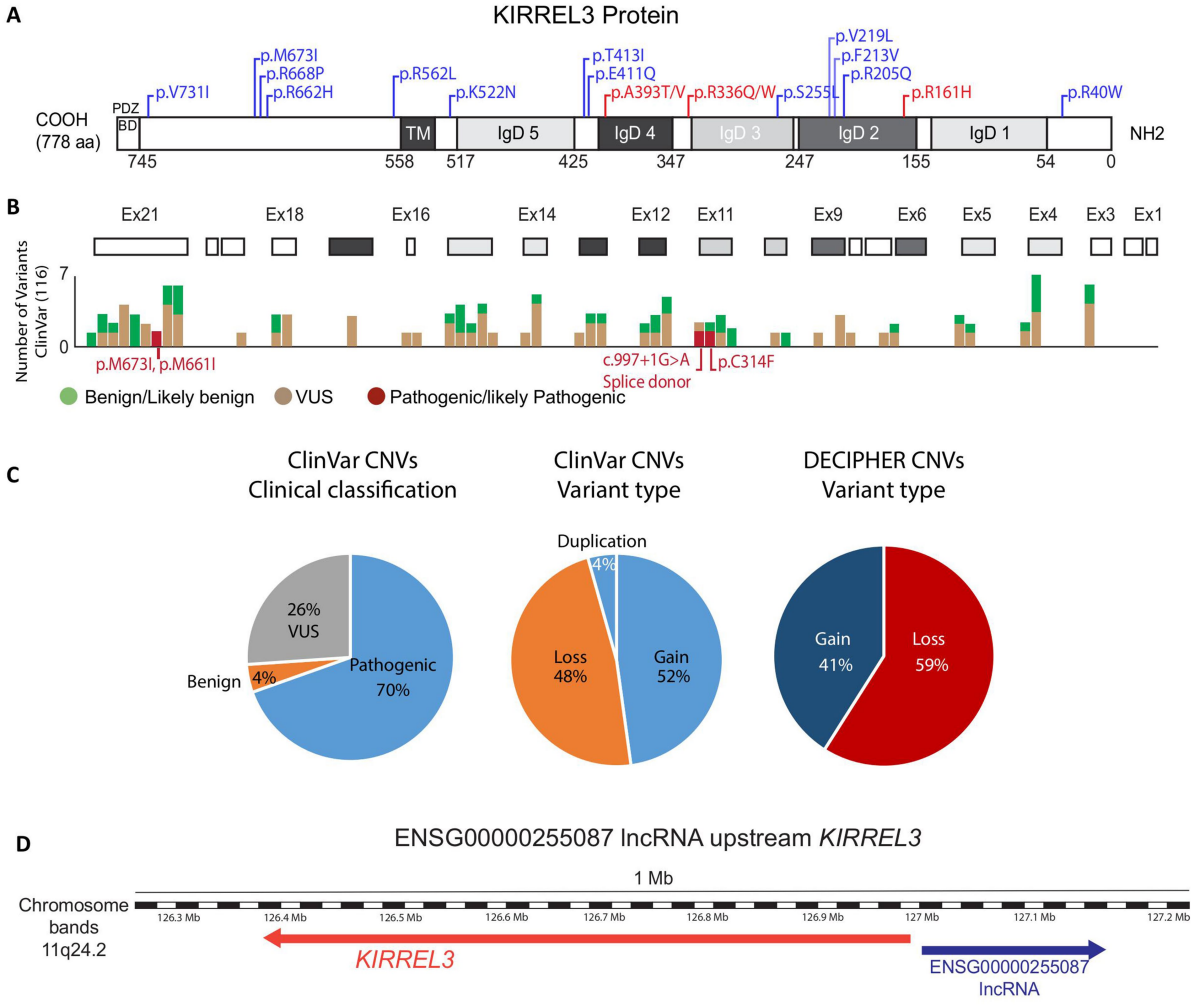
*KIRREL3* clinical variants. **(A)** KIRREL3 protein domains as predicted by SMART and UniProtKB/TrEMBL. Numbers in the bottom are amino acid (aa) number order. Domains are grey shaded to match their corresponding exons in **(B)**. Well-reported variants with detailed clinical manifestations are indicated on the protein domains, see Table 2. Variants with 1 or 2 occurrences are blue, variants with ≥3 occurrence are red. **(B)** Exons (boxes) are aligned with clinical variants reported on ClinVar. **(C)** Statistical counts on clinical variants types reported in ClinVar and DECIPHER. **(D)** Genetic map of *KIRREL3* and *Kirrel3-IncRNA*. IncRNA’s associated mutation was reported in an ASD patient as a potential regulator of the gene. See Methods for details. IgD, immunoglobulin domain; TM, transmembrane; BD, binding domain; VUS, variant of uncertain significance.

This analysis reveals two main points. First, variants are distributed across the entire protein (Figures 4A,B), with no obvious clustering toward specific domains. Second, most variants (56%) remain classified as variants of uncertain significance (VUS), with only 2% currently designated as pathogenic on ClinVar. Even functionally tested variants like p.R40W and p.V731F remain classified as VUS, despite evidence that they impair synapse formation in mice (2). This conservative approach suggests current guidelines may underestimate *KIRREL3*’s clinical importance.

We also searched for genetic evidence to establish *KIRREL3* as a risk factor for neurological disorders in large-scale studies on autism spectrum disorder and intellectual disability (FDR < 0.1) (2, 3, 5, 31, 75–84). From these studies, we curated 55 exonic SNVs that are well-phenotyped, and the connections are supported by computational predictions showing that many exonic missense variants have high pathogenicity scores (CADD ≥30), indicating strong likelihood of deleterious effects (Table 1) (85). Furthermore, we found that there is a predominance of *de novo* mutations, with equal maternal and paternal inheritance (75, 76, 80, 81, 86). Interestingly, the extracellular region has three recurrent pathogenic variants, each observed in ≥3 unrelated cases and associated with severe phenotypes, p.R161H, p.R336Q/W, p.A393T/V (Figure 4A in red, Table 2). Table 2 highlights the variants with reported detailed clinical manifestations. Interestingly, both extracellular variants, p.R161H and p.R336Q, disrupt synapse induction in mice (2). The 19 intracellular variants remain poorly characterized beyond the PDZ-binding domain’s putative synaptic scaffolding role (2, 10, 83). The recurrence of pathogenic extracellular variants exhibiting similar severe phenotypes suggests their clinical significance, warranting prioritization in genetic testing. Intracellular variants remain less understood but may contribute to variable expressivity. Future work should address the intracellular domain’s unexplored contributions to *KIRREL3*-related pathogenesis through structural biology and proteomic approaches. The combination of domain-wide variant distribution, functional evidence, and genetic associations supports *KIRREL3* as a clinically significant risk gene in neurological disorders. Improved variant interpretation methods are needed to fully capture the variants’ pathogenic potential.

### CNVs in clinical cases

*KIRREL3* deletions are a consistent feature of Jacobsen syndrome (chromosome 11q deletion syndrome), which typically results from large (7–20 Mb) terminal deletions at 11q23.3-qter that encompass *KIRREL3* (11q24.2) (34, 36, 87). Jacobsen syndrome is a rare genetic disorder characterized by developmental delays, craniofacial dysmorphisms, and variable congenital anomalies. The syndrome’s characteristic intellectual disability appears specifically linked to loss of *KIRREL3* gene. This is because two unusual cases with microdeletions (∼700 kb) sparing *KIRREL3* presented without intellectual disability (88). This genotype–phenotype correlation strongly suggests *KIRREL3* loss is the key contributor to cognitive impairment in Jacobsen syndrome.

We also analyzed other publicly available CNVs in ClinVar and DECIPHER in the same way we analyzed the SNVs in the previous section. *KIRREL3* CNVs show significantly high pathogenicity rates, with nearly equal proportions of duplications and deletions (gain and loss) (Figure 4C). Furthermore, the gene’s exceptionally high probability of loss-of-function intolerance (pLI = 0.98), suggests haploinsufficiency as a key disease mechanism in humans (74–76, 80). However, critical gaps remain in correlating these genetic findings with detailed clinical outcomes, as most databases lack comprehensive phenotypic information.

Beyond CNVs that disrupt KIRREL3’s protein-coding sequence, regulatory mechanisms may also contribute to *KIRREL3*-related neurological disorders. A study of chromosomal abnormalities in genetically undiagnosed neurological cases identified a patient with ADHD and spatial coordination deficits carrying *de novo* balanced breakpoints at 11q24.2 (89, 90). While no pathogenic coding variants were detected, the breakpoints disrupted *ENSG00000255087*, a long non-coding RNA (lncRNA) located upstream of *KIRREL3* (Figure 4D). Subsequent analysis demonstrated co-occurring *KIRREL3* mRNA downregulation in this patient. This suggests that either the lncRNA regulates *KIRREL3* gene expression or this region contains an enhancer regulatory element for *KIRREL3* gene. These findings highlight the importance of investigating nearby regulatory element variants where non-coding disruptions can phenocopy coding mutations through transcriptional dysregulation.

## Future perspectives

Moving forward, research on *KIRREL3* should address several critical gaps to fully understand its role in neural circuit development and neurological disorders. While studies revealed essential *Kirrel3* functions in hippocampal and olfactory systems, conditional knockout approaches targeting specific neuronal populations will be necessary to dissect its roles in cortical, cerebellar, and other neural circuits. The existence of multiple *Kirrel3* isoforms, particularly the distinct secreted and transmembrane forms, presents another crucial area for exploration. Detailed characterization of isoform-specific localization, binding partners, and functional differences could explain the phenotypic variability observed in patients and provide mechanistic insights into how different mutations lead to diverse clinical outcomes.

There are currently no published studies on human iPSC-derived neurons from patients with *KIRREL3* mutations. However, human cellular models will be essential for translating findings from animal studies to clinical applications. Patient-derived iPSC neurons with defined *KIRREL3* variants offer a powerful platform to validate mechanisms observed in mice while capturing human-specific aspects of neural development. On the clinical front, establishing standardized phenotyping and reporting protocols across clinic centers will be critical for capturing the full spectrum of *KIRREL3*-related manifestations and correlating them with specific genetic changes.

These future perspectives require collaboration between neurobiologists studying synaptic development, geneticists characterizing variants, and clinicians caring for affected individuals. In conclusion, *KIRREL3* emerges as a model for understanding how synaptic adhesion molecules contribute to neurological disorders. Its complex isoform regulation, region-specific circuit functions, and diverse mutational mechanisms illustrate the intricate relationship between molecular neurobiology and clinical presentation.

## References

[1] Fischbach KF Linneweber GA Felix Malte Andlauer T Hertenstein A Bonengel B Chaudhary K. The irre cell recognition module (IRM) proteins. J Neurogenet. (2009) 23:48–67. doi: 10.1080/01677060802471668, PMID: 19132596

[2] Taylor MR Martin EA Sinnen B Trilokekar R Ranza E Antonarakis SE. Kirrel3-mediated synapse formation is attenuated by disease-associated missense variants. J Neurosci. (2020) 40:5376–88. doi: 10.1523/JNEUROSCI.3058-19.2020, PMID: 32503885 PMC7343328

[3] Querzani A Sirchia F Rustioni G Rossi A Orsini A Marseglia GL. KIRREL3-related disorders: a case report confirming the radiological features and expanding the clinical spectrum to a less severe phenotype. Ital J Pediatr. (2023) 49:99. doi: 10.1186/s13052-023-01488-7, PMID: 37605258 PMC10441694

[4] Guerin A Stavropoulos DJ Diab Y Chénier S Christensen H Kahr WHA. Interstitial deletion of 11q-implicating the KIRREL3 gene in the neurocognitive delay associated with Jacobsen syndrome. Am J Med Genet A. (2012) 158a:2551–6. doi: 10.1002/ajmg.a.35621, PMID: 22965935

[5] Kalsner L Twachtman-Bassett J Tokarski K Stanley C Dumont-Mathieu T Cotney J. Genetic testing including targeted gene panel in a diverse clinical population of children with autism spectrum disorder: findings and implications. Mol Genet Genomic Med. (2018) 6:171–85. doi: 10.1002/mgg3.354, PMID: 29271092 PMC5902398

[6] Neumann-Haefelin E Kramer-Zucker A Slanchev K Hartleben B Noutsou F Martin K. A model organism approach: defining the role of Neph proteins as regulators of neuron and kidney morphogenesis. Hum Mol Genet. (2010) 19:2347–59. doi: 10.1093/hmg/ddq108, PMID: 20233749

[7] Durcan PJ Al-Shanti N Stewart CE. Identification and characterization of novel Kirrel isoform during myogenesis. Physiol Rep. (2013) 1:e00044. doi: 10.1002/phy2.44, PMID: 24303129 PMC3835000

[8] Donoviel DB Freed DD Vogel H Potter DG Hawkins E Barrish JP. Proteinuria and perinatal lethality in mice lacking NEPH1, a novel protein with homology to NEPHRIN. Mol Cell Biol. (2001) 21:4829–36. doi: 10.1128/MCB.21.14.4829-4836.2001, PMID: 11416156 PMC87176

[9] George B Holzman LB. Signaling from the podocyte intercellular junction to the actin cytoskeleton. Semin Nephrol. (2012) 32:307–18. doi: 10.1016/j.semnephrol.2012.06.002, PMID: 22958485 PMC3438455

[10] Liu YF Sowell SM Luo Y Chaubey A Cameron RS Kim HG. Autism and intellectual disability-associated KIRREL3 interacts with neuronal proteins MAP1B and MYO16 with potential roles in neurodevelopment. PLoS One. (2015) 10:e0123106. doi: 10.1371/journal.pone.0123106, PMID: 25902260 PMC4406691

[11] Chao DL Shen K. Functional dissection of SYG-1 and SYG-2, cell adhesion molecules required for selective synaptogenesis in *C. elegans*. Mol Cell Neurosci. (2008) 39:248–57. doi: 10.1016/j.mcn.2008.07.001, PMID: 18675916 PMC2575880

[12] Wanner N Noutsou F Baumeister R Walz G Huber TB Neumann-Haefelin E. Functional and spatial analysis of *C. elegans* SYG-1 and SYG-2, orthologs of the Neph/nephrin cell adhesion module directing selective synaptogenesis. PLoS One. (2011) 6:e23598. doi: 10.1371/journal.pone.0023598, PMID: 21858180 PMC3156230

[13] Shen K Bargmann CI. The immunoglobulin superfamily protein SYG-1 determines the location of specific synapses in *C. elegans*. Cell. (2003) 112:619–30. doi: 10.1016/S0092-8674(03)00113-2, PMID: 12628183

[14] Srinivas BP Woo J Leong WY Roy S. A conserved molecular pathway mediates myoblast fusion in insects and vertebrates. Nat Genet. (2007) 39:781–6. doi: 10.1038/ng2055, PMID: 17529975

[15] Tamir-Livne Y Mubariki R Bengal E. Adhesion molecule Kirrel3/Neph2 is required for the elongated shape of myocytes during skeletal muscle differentiation. Int J Dev Biol. (2017) 61:337–45. doi: 10.1387/ijdb.170005eb, PMID: 28621431

[16] Durcan PJ Conradie JD Van deVyver M Myburgh KH. Identification of novel Kirrel3 gene splice variants in adult human skeletal muscle. BMC Physiol. (2014) 14:11. doi: 10.1186/s12899-014-0011-3, PMID: 25488023 PMC4269076

[17] Ristola M Lehtonen S. Functions of the podocyte proteins nephrin and Neph3 and the transcriptional regulation of their genes. Clin Sci (Lond). (2014) 126:315–28. doi: 10.1042/CS20130258, PMID: 24219158

[18] Yang B Zhang X Zhou H Zhang X Yang W Lu J. Preliminary study on the role and mechanism of KIRREL3 in the development of esophageal squamous cell carcinoma. Pathol Res Pract. (2022) 237:154025. doi: 10.1016/j.prp.2022.154025, PMID: 35863131

[19] Lüthy K Ahrens B Rawal S Lu Z Tarnogorska D Meinertzhagen IA. The irre cell recognition module (IRM) protein Kirre is required to form the reciprocal synaptic network of L4 neurons in the Drosophila lamina. J Neurogenet. (2014) 28:291–301. doi: 10.3109/01677063.2014.883390, PMID: 24697410

[20] Zhuang S Shao H Guo F Trimble R Pearce E Abmayr SM. Sns and Kirre, the Drosophila orthologs of Nephrin and Neph1, direct adhesion, fusion and formation of a slit diaphragm-like structure in insect nephrocytes. Development. (2009) 136:2335–44. doi: 10.1242/dev.031609, PMID: 19515699 PMC2729346

[21] Tucker DK Adams CS Prasad G Ackley BD. The immunoglobulin superfamily members syg-2 and syg-1 regulate neurite development in *C. elegans*. J Dev Biol. (2022) 10:1003. doi: 10.3390/jdb10010003, PMID: 35076532 PMC8788504

[22] Strünkelnberg M Bonengel B Moda LM Hertenstein A de Couet HG Ramos RG. Rst and its paralogue kirre act redundantly during embryonic muscle development in Drosophila. Development. (2001) 128:4229–39. doi: 10.1242/dev.128.21.4229, PMID: 11684659

[23] Shen K Fetter RD Bargmann CI. Synaptic specificity is generated by the synaptic guidepost protein SYG-2 and its receptor, SYG-1. Cell. (2004) 116:869–81. doi: 10.1016/S0092-8674(04)00251-X, PMID: 15035988

[24] Özkan E Chia PH Wang RR Goriatcheva N Borek D Otwinowski Z. Extracellular architecture of the SYG-1/SYG-2 adhesion complex instructs synaptogenesis. Cell. (2014) 156:482–94. doi: 10.1016/j.cell.2014.01.004, PMID: 24485456 PMC3962013

[25] Machado MC Octacilio-Silva S Costa MS Ramos RG. Rst transcriptional activity influences kirre mRNA concentration in the Drosophila pupal retina during the final steps of ommatidial patterning. PLoS One. (2011) 6:e22536. doi: 10.1371/journal.pone.0022536, PMID: 21857931 PMC3152562

[26] Chia PH Chen B Li P Rosen MK Shen K. Local F-actin network links synapse formation and axon branching. Cell. (2014) 156:208–20. doi: 10.1016/j.cell.2013.12.009, PMID: 24439377 PMC3954643

[27] Bao S Fischbach KF Corbin V Cagan RL. Preferential adhesion maintains separation of ommatidia in the Drosophila eye. Dev Biol. (2010) 344:948–56. doi: 10.1016/j.ydbio.2010.06.013, PMID: 20599904 PMC2921583

[28] Helmstädter M Lüthy K Gödel M Simons M Ashish Nihalani D. Functional study of mammalian Neph proteins in *Drosophila melanogaster*. PLoS One. (2012) 7:e40300. doi: 10.1371/journal.pone.0040300, PMID: 22792268 PMC3391254

[29] Haralalka S Shelton C Cartwright HN Guo F Trimble R Kumar RP. Live imaging provides new insights on dynamic F-actin filopodia and differential endocytosis during myoblast fusion in Drosophila. PLoS One. (2014) 9:e114126. doi: 10.1371/journal.pone.0114126, PMID: 25474591 PMC4256407

[30] Helmstädter M Höhne M Huber TB. A brief overview on IRM function across evolution. J Neurogenet. (2014) 28:264–9. doi: 10.3109/01677063.2014.918976, PMID: 24912528

[31] Bhalla K Luo Y Buchan T Beachem MA Guzauskas GF Ladd S. Alterations in CDH15 and KIRREL3 in patients with mild to severe intellectual disability. Am J Hum Genet. (2008) 83:703–13. doi: 10.1016/j.ajhg.2008.10.020, PMID: 19012874 PMC2668064

[32] Kotian N Troike KM Curran KN Lathia JD McDonald JA. A drosophila RNAi screen reveals conserved glioblastoma-related adhesion genes that regulate collective cell migration. G3. (2021) 12:356. doi: 10.1093/g3journal/jkab356, PMID: 34849760 PMC8728034

[33] Yi GZ Zhang HY Que TS Qu SQ Li ZY Qi ST. Identification of the clinical and genetic characteristics of gliomas with gene fusions by integrated genomic and transcriptomic analysis. Eur J Med Res. (2025) 30:49. doi: 10.1186/s40001-025-02306-y, PMID: 39849652 PMC11755825

[34] Serra G Memo L Antona V Corsello G Favero V Lago P. Jacobsen syndrome and neonatal bleeding: report on two unrelated patients. Ital J Pediatr. (2021) 47:147. doi: 10.1186/s13052-021-01108-2, PMID: 34210338 PMC8252210

[35] Linares Chávez EP Toral López J Valdés Miranda JM González Huerta LM Perez Cabrera A del Refugio Rivera Vega M. Jacobsen syndrome: surgical complications due to unsuspected diagnosis, the importance of molecular studies in patients with Craniosynostosis. Mol Syndromol. (2016) 6:229–35. doi: 10.1159/000442477, PMID: 26997943 PMC4772712

[36] Anzick S Thurm A Burkett S Velez D Cho E Chlebowski C. Chromoanasynthesis as a cause of Jacobsen syndrome. Am J Med Genet A. (2020) 182:2533–9. doi: 10.1002/ajmg.a.61824, PMID: 32841469 PMC11007684

[37] Baccarin M Picinelli C Tomaiuolo P Castronovo P Costa A Verdecchia M. Appropriateness of array-CGH in the ADHD clinics: a comparative study. Genes Brain Behav. (2020) 19:e12651. doi: 10.1111/gbb.12651, PMID: 32141190

[38] Collins RL Brand H Karczewski KJ Zhao X Alföldi J Francioli LC. A structural variation reference for medical and population genetics. Nature. (2020) 581:444–51. doi: 10.1038/s41586-020-2287-8, PMID: 32461652 PMC7334194

[39] Perez G Barber GP Benet-Pages A Casper J Clawson H Diekhans M. The UCSC genome browser database: 2025 update. Nucleic Acids Res. (2025) 53:D1243–d1249. doi: 10.1093/nar/gkae974, PMID: 39460617 PMC11701590

[40] Belmadani M Jacobson M Holmes N Phan M Nguyen T Pavlidis P. VariCarta: a comprehensive database of harmonized genomic variants found in autism Spectrum disorder sequencing studies. Autism Res. (2019) 12:1728–36. doi: 10.1002/aur.2236, PMID: 31705629

[41] Landrum MJ Lee JM Benson M Brown GR Chao C Chitipiralla S. ClinVar: improving access to variant interpretations and supporting evidence. Nucleic Acids Res. (2018) 46:D1062–d1067. doi: 10.1093/nar/gkx1153, PMID: 29165669 PMC5753237

[42] Makrilia N Kollias A Manolopoulos L Syrigos K. Cell adhesion molecules: role and clinical significance in cancer. Cancer Investig. (2009) 27:1023–37. doi: 10.3109/07357900902769749, PMID: 19909018

[43] Sellin L Huber TB Gerke P Quack I Pavenstädt H Walz G. NEPH1 defines a novel family of podocin interacting proteins. FASEB J 17:115–7. doi: 10.1096/fj.02-0242fje 12424224

[44] Dyer SC Austine-Orimoloye O Azov AG Barba M Barnes I Barrera-Enriquez VP. Ensembl 2025. Nucleic Acids Res. (2025) 53:D948–d957. doi: 10.1093/nar/gkae1071, PMID: 39656687 PMC11701638

[45] Traenkner D. Modular splicing is linked to evolution in the synapse-specificity molecule Kirrel3. eNeuro. (2023) 10:ENEURO.0253-0223.2023. doi: 10.1523/ENEURO.0253-23.2023 PMC10698715 37977826

[46] Siletti K Hodge R Mossi Albiach A Lee KW Ding SL Hu L. Transcriptomic diversity of cell types across the adult human brain. Science. (2023) 382:eadd7046. doi: 10.1126/science.add7046, PMID: 37824663

[47] Tamura S Morikawa Y Hisaoka T Ueno H Kitamura T Senba E. Expression of mKirre, a mammalian homolog of Drosophila kirre, in the developing and adult mouse brain. Neuroscience. (2005) 133:615–24. doi: 10.1016/j.neuroscience.2005.03.030, PMID: 15908127

[48] Serizawa S Miyamichi K Takeuchi H Yamagishi Y Suzuki M Sakano H. A neuronal identity code for the odorant receptor-specific and activity-dependent axon sorting. Cell. (2006) 127:1057–69. doi: 10.1016/j.cell.2006.10.031, PMID: 17129788

[49] Yao Z van Velthoven CTJ Nguyen TN Goldy J Sedeno-Cortes AE Baftizadeh F. A taxonomy of transcriptomic cell types across the isocortex and hippocampal formation. Cell. (2021) 184:3222–3241.e26. doi: 10.1016/j.cell.2021.04.021, PMID: 34004146 PMC8195859

[50] Wu C Jin X Tsueng G Afrasiabi C Su AI. BioGPS: building your own mash-up of gene annotations and expression profiles. Nucleic Acids Res. (2016) 44:D313–6. doi: 10.1093/nar/gkv1104, PMID: 26578587 PMC4702805

[51] Karlsson M Zhang C Méar L Zhong W Digre A Katona B. A single-cell type transcriptomics map of human tissues. Sci Adv. (2021) 7:169. doi: 10.1126/sciadv.abh2169, PMID: 34321199 PMC8318366

[52] Martin EA Muralidhar S Wang Z Cervantes DC Basu R Taylor MR. Correction: the intellectual disability gene Kirrel3 regulates target-specific mossy fiber synapse development in the hippocampus. eLife. (2016) 5:18706. doi: 10.7554/eLife.18706, PMID: 27310701 PMC4911213

[53] Breschi A Gingeras TR Guigó R. Comparative transcriptomics in human and mouse. Nat Rev Genet. (2017) 18:425–40. doi: 10.1038/nrg.2017.19, PMID: 28479595 PMC6413734

[54] Monaco G van Dam S Casal Novo Ribeiro JL Larbi A de Magalhães JP. A comparison of human and mouse gene co-expression networks reveals conservation and divergence at the tissue, pathway and disease levels. BMC Evol Biol. (2015) 15:259. doi: 10.1186/s12862-015-0534-7, PMID: 26589719 PMC4654840

[55] Su AI Cooke MP Ching KA Hakak Y Walker JR Wiltshire T. Large-scale analysis of the human and mouse transcriptomes. Proc Natl Acad Sci USA. (2002) 99:4465–70. doi: 10.1073/pnas.012025199, PMID: 11904358 PMC123671

[56] Hisaoka T Komori T Kitamura T Morikawa Y. Abnormal behaviours relevant to neurodevelopmental disorders in Kirrel3-knockout mice. Sci Rep. (2018) 8:1408. doi: 10.1038/s41598-018-19844-7, PMID: 29362445 PMC5780462

[57] Völker LA Maar BA Pulido Guevara BA Bilkei-Gorzo A Zimmer A Brönneke H. Neph2/Kirrel3 regulates sensory input, motor coordination, and home-cage activity in rodents. Genes Brain Behav. (2018) 17:e12516. doi: 10.1111/gbb.12516, PMID: 30133126

[58] Choi SY Han K Cutforth T Chung W Park H Lee D. Mice lacking the synaptic adhesion molecule Neph2/Kirrel3 display moderate hyperactivity and defective novel object preference. Front Cell Neurosci. (2015) 9:283. doi: 10.3389/fncel.2015.00283, PMID: 26283919 PMC4517382

[59] Tuñon-Ortiz A Tränkner D Peterson CM Shennib O Ye F Shi J. Inhibitory neurons marked by the connectivity molecule Kirrel3 regulate memory precision. J Neurosci. (2025):e1760242025. doi: 10.1523/JNEUROSCI.1760-24.2025, PMID: 40769722 PMC12444912

[60] Prince JE Brignall AC Cutforth T Shen K Cloutier JF. Kirrel3 is required for the coalescence of vomeronasal sensory neuron axons into glomeruli and for male-male aggression. Development. (2013) 140:2398–408. doi: 10.1242/dev.087262, PMID: 23637329 PMC3653560

[61] Völker LA Petry M Abdelsabour-Khalaf M Schweizer H Yusuf F Busch T. Comparative analysis of Neph gene expression in mouse and chicken development. Histochem Cell Biol. (2012) 137:355–66. doi: 10.1007/s00418-011-0903-2, PMID: 22205279 PMC3278613

[62] Danesh AA Howery S Aazh H Kaf W Eshraghi AA. Hyperacusis in autism Spectrum disorders. Audiol Res. (2021) 11:547–56. doi: 10.3390/audiolres11040049, PMID: 34698068 PMC8544234

[63] Amaral DG Schumann CM Nordahl CW. Neuroanatomy of autism. Trends Neurosci. (2008) 31:137–45. doi: 10.1016/j.tins.2007.12.005, PMID: 18258309

[64] Hazen EP Stornelli JL O'Rourke JA Koesterer K McDougle CJ. Sensory symptoms in autism spectrum disorders. Harv Rev Psychiatry. (2014) 22:112–24. doi: 10.1097/01.HRP.0000445143.08773.58, PMID: 24614766

[65] Roh JD Choi SY Cho YS Choi TY Park JS Cutforth T. Increased excitatory synaptic transmission of dentate granule neurons in mice lacking PSD-95-interacting adhesion molecule Neph2/Kirrel3 during the early postnatal period. Front Mol Neurosci. (2017) 10:81. doi: 10.3389/fnmol.2017.00081, PMID: 28381988 PMC5360738

[66] Martin EA Woodruff D Rawson RL Williams ME. Examining hippocampal mossy fiber synapses by 3D electron microscopy in wildtype and Kirrel3 knockout mice. eNeuro. (2017) 4:88. doi: 10.1523/ENEURO.0088-17.2017, PMID: 28670619 PMC5490256

[67] Bourgeron T. From the genetic architecture to synaptic plasticity in autism spectrum disorder. Nat Rev Neurosci. (2015) 16:551–63. doi: 10.1038/nrn3992, PMID: 26289574

[68] Zhao H Mao X Zhu C Zou X Peng F Yang W. GABAergic system dysfunction in autism spectrum disorders. Front Cell Dev Biol. (2021) 9:781327. doi: 10.3389/fcell.2021.781327, PMID: 35198562 PMC8858939

[69] Morikawa Y Komori T Hisaoka T Ueno H Kitamura T Senba E. Expression of mKirre in the developing sensory pathways: its close apposition to nephrin-expressing cells. Neuroscience. (2007) 150:880–6. doi: 10.1016/j.neuroscience.2007.10.013, PMID: 18022324

[70] Imai T Sakano H. Axon-axon interactions in neuronal circuit assembly: lessons from olfactory map formation. Eur J Neurosci. (2011) 34:1647–54. doi: 10.1111/j.1460-9568.2011.07817.x, PMID: 22103421

[71] Wang J Vaddadi N Pak JS Park Y Quilez S Roman CA. Molecular and structural basis of olfactory sensory neuron axon coalescence by Kirrel receptors. Cell Rep. (2021) 37:109940. doi: 10.1016/j.celrep.2021.109940, PMID: 34731636 PMC8628261

[72] Brusés JL. Identification of gene transcripts expressed by postsynaptic neurons during synapse formation encoding cell surface proteins with presumptive synaptogenic activity. Synapse. (2010) 64:47–60. doi: 10.1002/syn.20702, PMID: 19728367 PMC2783745

[73] Grossfeld PD Mattina T Lai Z Favier R Jones KL Cotter F. The 11q terminal deletion disorder: a prospective study of 110 cases. Am J Med Genet A. (2004) 129a:51–61. doi: 10.1002/ajmg.a.30090, PMID: 15266616

[74] Karczewski KJ Francioli LC Tiao G Cummings BB Alföldi J Wang Q. The mutational constraint spectrum quantified from variation in 141,456 humans. Nature. (2020) 581:434–43. doi: 10.1038/s41586-020-2308-7, PMID: 32461654 PMC7334197

[75] Zhou X Feliciano P Shu C Wang T Astrovskaya I Hall JB. Integrating de novo and inherited variants in 42,607 autism cases identifies mutations in new moderate-risk genes. Nat Genet. (2022) 54:1305–19. doi: 10.1038/s41588-022-01148-2, PMID: 35982159 PMC9470534

[76] Trost B Thiruvahindrapuram B Chan AJS Engchuan W Higginbotham EJ Howe JL. Genomic architecture of autism from comprehensive whole-genome sequence annotation. Cell. (2022) 185:4409–4427.e18. doi: 10.1016/j.cell.2022.10.009, PMID: 36368308 PMC10726699

[77] Li J Wang L Guo H Shi L Zhang K Tang M. Targeted sequencing and functional analysis reveal brain-size-related genes and their networks in autism spectrum disorders. Mol Psychiatry. (2017) 22:1282–90. doi: 10.1038/mp.2017.140, PMID: 28831199

[78] De Rubeis S He X Goldberg AP Poultney CS Samocha K Cicek AE. Synaptic, transcriptional and chromatin genes disrupted in autism. Nature. (2014) 515:209–15. doi: 10.1038/nature13772, PMID: 25363760 PMC4402723

[79] Hu C Wang Y Li C Mei L Zhou B Li D. Targeted sequencing and clinical strategies in children with autism spectrum disorder: a cohort study. Front Genet. (2023) 14:1083779. doi: 10.3389/fgene.2023.1083779, PMID: 37007974 PMC10064793

[80] Stessman HA Xiong B Coe BP Wang T Hoekzema K Fenckova M. Targeted sequencing identifies 91 neurodevelopmental-disorder risk genes with autism and developmental-disability biases. Nat Genet. (2017) 49:515–26. doi: 10.1038/ng.3792, PMID: 28191889 PMC5374041

[81] Guo H Wang T Wu H Long M Coe BP Li H. Inherited and multiple de novo mutations in autism/developmental delay risk genes suggest a multifactorial model. Mol Autism. (2018) 9:64. doi: 10.1186/s13229-018-0247-z, PMID: 30564305 PMC6293633

[82] Leblond CS Cliquet F Carton C Huguet G Mathieu A Kergrohen T. Both rare and common genetic variants contribute to autism in the Faroe Islands. NPJ Genom Med. (2019) 4:1. doi: 10.1038/s41525-018-0075-2, PMID: 30675382 PMC6341098

[83] Ciaccio C Leonardi E Polli R Murgia A D'Arrigo S Granocchio E. A missense De Novo variant in the CASK-interactor KIRREL3 gene leading to neurodevelopmental disorder with mild cerebellar hypoplasia. Neuropediatrics. (2021) 52:484–8. doi: 10.1055/s-0041-1725964, PMID: 33853164

[84] Hildebrand MS Jackson VE Scerri TS van Reyk O Coleman M Braden RO. Severe childhood speech disorder: gene discovery highlights transcriptional dysregulation. Neurology. (2020) 94:e2148–67. doi: 10.1212/WNL.0000000000009441, PMID: 32345733

[85] Kircher M Witten DM Jain P O'Roak BJ Cooper GM Shendure J. A general framework for estimating the relative pathogenicity of human genetic variants. Nat Genet. (2014) 46:310–5. doi: 10.1038/ng.2892, PMID: 24487276 PMC3992975

[86] Deciphering Developmental Disorders Study. Prevalence and architecture of de novo mutations in developmental disorders. Nature. (2017) 542:433–8. doi: 10.1038/nature21062, PMID: 28135719 PMC6016744

[87] Mattina T Perrotta CS Grossfeld P. Jacobsen syndrome. Orphanet J Rare Dis. (2009) 4:9. doi: 10.1186/1750-1172-4-9, PMID: 19267933 PMC2670819

[88] Conrad S Demurger F Moradkhani K Pichon O le Caignec C Pascal C. 11q24.2q24.3 microdeletion in two families presenting features of Jacobsen syndrome, without intellectual disability: role of FLI1, ETS1, and SENCR long noncoding RNA. Am J Med Genet A. (2019) 179:993–1000. doi: 10.1002/ajmg.a.61113, PMID: 30888095

[89] Talkowski ME Rosenfeld JA Blumenthal I Pillalamarri V Chiang C Heilbut A. Sequencing chromosomal abnormalities reveals neurodevelopmental loci that confer risk across diagnostic boundaries. Cell. (2012) 149:525–37. doi: 10.1016/j.cell.2012.03.028, PMID: 22521361 PMC3340505

[90] Andersen RE Alkuraya IF Ajeesh A Sakamoto T Mena EL Amr SS. Chromosomal structural rearrangements implicate long non-coding RNAs in rare germline disorders. Hum Genet. (2024) 143:921–38. doi: 10.1007/s00439-024-02693-y, PMID: 39060644 PMC11294402

[91] Guo H Duyzend MH Coe BP Baker C Hoekzema K Gerdts J. Genome sequencing identifies multiple deleterious variants in autism patients with more severe phenotypes. Genet Med. (2019) 21:1611–1620. doi: 10.1038/s41436-018-0380-2, PMID: 30504930 PMC6546556

[92] Xin J Mark A Afrasiabi C Tsueng G Juchler M Gopal N. High-performance web services for querying gene and variant annotation. Genome Biol. (2016) 17:91. doi: 10.1186/s13059-016-0953-9 27154141 PMC4858870

